# The Diagnosis of Invasive and Noninvasive Pulmonary Aspergillosis by Serum and Bronchoalveolar Lavage Fluid Galactomannan Assay

**DOI:** 10.1155/2015/943691

**Published:** 2015-01-22

**Authors:** Shuzhen Zhang, Sibu Wang, Zhe Wan, Ruoyu Li, Jin Yu

**Affiliations:** Department of Dermatology and Venereology, Peking University First Hospital, Research Center for Medical Mycology, Peking University, Beijing 100034, China

## Abstract

The incidence and mortality of invasive pulmonary aspergillosis (IPA) are rising, particularly in critically ill patients and patients with severe chronic obstructive pulmonary disease (COPD). Noninvasive aspergillosis occurring in these patients requires special attention because of the possibility of developing subsequent IPA, given the poor health and worsened immune state of these patients. We compared the performance of the Platelia galactomannan (GM) enzyme immunoassay in the bronchoalveolar lavage fluid (BALF) and serum. The sensitivity, and specificity of BALF-GM were 85.4% and 62.4%, and those of serum-GM were 67.9% and 93.5% at the cutoff index of 0.5. As the cutoff index increased, the specificity of BALF-GM detection was increased with the detriment of sensitivity. The area under the ROC curves was 0.817 (95% CI: 0.718–0.916) for BALF-GM and 0.819 (95% CI: 0.712–0.926) for serum-GM. The optimal cutoff index was 1.19 for BALF-GM, and the sensitivity and specificity were 67.9% and 89.2%. The BALF-GM assay is more sensitive in detecting pulmonary aspergillosis than serum-GM assay and fungal cultures. However, BALF-GM assay has a high false-positive rate at the cutoff index of 0.5. Hence, the diagnostic cutoff index of the BALF-GM assay should be improved to avoid the overdiagnosis of pulmonary aspergillosis in clinic.

## 1. Introduction

*Aspergillus* is a saprophytic fungus of ubiquitous occurrence in our living environment. People inevitably inhale the spores or conidia every day, which can induce various types of clinical conditions including invasive pulmonary aspergillosis (IPA), chronic necrotizing aspergillosis (CNA), allergic bronchopulmonary aspergillosis (ABPA), and aspergilloma [1].

IPA, the most severe type of pulmonary aspergillosis, is a significant cause of mortality in immunocompromised individuals, especially in those with malignant hematological disorders such as neutropenia and hematopoietic stem cell transplantation. The incidence and mortality of IPA are rising [2]. Notably, the incidence of invasive aspergillosis in critically ill patients and patients with severe chronic obstructive pulmonary disease (COPD) has increased in recent years. Patients with invasive aspergillosis have a high mortality and are difficult to early diagnose; hence, sensitive assays for accurate diagnosis of the fungal infection are warranted [3–6].

CNA is a localized pulmonary lesion caused by* Aspergillus* spp. and invariably has a chronic clinical course. The infection is common in patients with underlying pulmonary diseases, such as COPD, pulmonary cystic fibrosis, old pulmonary tuberculosis, and silicosis, or in patients with poor immunological function, such as alcoholics, diabetics, and the malnourished. Although CNA is not as severe as invasive pulmonary disease, it still has a high mortality rate of 40%–60% [4]. ABPA is an allergic immunological response caused by* Aspergillus *spp. and has a clinical manifestation similar to asthma. Aspergilloma is a fungal ball formed as a result of* Aspergillus* spp. colonization and growth, leading to destructive pulmonary diseases, such as pulmonary cavitation or bronchiolectasis. CNA and aspergilloma, but not ABPA, can exacerbate to an invasive type once a patient's immune function weakens.

Galactomannan (GM) is a universal polysaccharide component of the cell wall in the* Aspergillus* spp. and is released into the bronchoalveolar lavage fluid (BALF) and blood during growth. Therefore, the detection of the GM antigen in BALF and serum serves as a reliable assay for the diagnosis of pulmonary aspergillosis. A positive Platelia galactomannan (GM) enzyme immunoassay (EIA) has been approved as important criteria for the diagnosis of invasive aspergillosis by the European Organization for Research and Treatment of Cancer/Invasive Fungal Infections Cooperative Group and the National Institute of Allergy and Infectious Diseases Mycoses Study Group (EORTC/MSG) consensus group [7].

However, the level of GM released into the blood is relatively low and usually cannot be detected in the early stages of IPA or noninvasive aspergillosis when the fungal lesions on the vessels are mild. The probability of detecting galactomannan is higher in BALF, which is lavaged directly from the target organ affected by pulmonary aspergillosis. This, in turn, improves the sensitivity of GM EIA commonly used to diagnose pulmonary aspergillosis.

To compare the diagnostic values (sensitivities) of the BALF-GM assay and the serum-GM assay for pulmonary aspergillosis, we conducted a retrospective study of inpatients who gave consent for both BALF-GM and serum-GM assays.

## 2. Materials and Methods

### 2.1. Patients

Inpatients who consented for the BALF-GM and serum-GM assays between March 2011 and September 2012 at the Peking University First Hospital, China, were enrolled in this retrospective study. All patients were suspected of having pulmonary aspergillosis and tested for the BALF-GM assay and serum-GM assay. The volume of BALF obtained in our study was 100 mL, and the recovery rate ranged from 30% to 60%.

Clinical data including disease history, clinical manifestations, diagnostic results, and treatment was collected for every patient.

### 2.2. GM Assay

To aid clinical diagnosis, GM levels were detected by using the Platelia Aspergillus EIA (Bio-Rad, Marnes la Coquette, France). The BALF-GM and serum-GM assays were performed in accordance with the manufacturer's specifications. The GM index was defined as the ratio of sample OD to control OD. The overall assay results were considered positive if the GM index was ≥0.5 for both serum-GM and BALF-GM results.

### 2.3. Case Definitions

Invasive pulmonary aspergillosis was diagnosed based on the revised definitions of invasive fungal disease from the EORTC/MSG [7]. Accordingly, the patients were diagnosed as having “proven,” “probable,” or “possible” IPA.

Proven IPA needs a positive histopathologic result or a positive culture of the tissue sample obtained from the target lesion by transthoracic needle biopsy or transbronchial lung biopsy. Conformation of probable disease requires specifics on host factor, clinical manifestations, and microbiology.

Those cases that possess the host and clinical factors, but lack any microbiological evidence, were classified as having possible IPA. Microbiological evidence includes the following criteria: (1) positive isolation of* Aspergillus* species from respiratory tract samples by culture; (2) at least two consecutive showing serum-GM positivity or BALF-GM positivity or both serum-GM positivity and BALF-GM positivity; (3) antibiotic treatment invalid, but antifungal treatment against aspergillosis valid. The microbiological evidence is acceptable with the presence of criteria (1), (3) or (2), (3) or (1), (2), and (3). The definition of noninvasive pulmonary aspergillosis was based on the diagnostic criteria used in previous reports [1].

The infectious group of patients included those with IPA, CNA, ABPA, and pulmonary aspergilloma, and the noninfectious group of patients included those with possible aspergillosis and inpatients in whom pulmonary aspergillosis was excluded.

### 2.4. Statistical Analysis

Sensitivity, specificity, positive predictive index (PPV), and negative predictive index (NPV) were calculated for fungal cultures and the BALF-GM and serum-GM assays. Additionally, the receiver operating characteristic curves (ROC) were constructed for the BALF-GM and serum-GM to acquire the optimal cutoff indices. Furthermore, factors causing false positive results in the BALF-GM assay were analyzed. The difference in GM indices of both the assays was analyzed using the Mann-Whitney test, and the categorical variables were listed in two-by-two contingency tables and analyzed using the Pearson's *χ* ^2^ test or Fisher test. A two-tailed *P* value of <0.05 was considered as statistically significant.

## 3. Results 

### 3.1. Patient Characteristics

A total of 121 inpatients were enrolled in our retrospective study. Patient's characteristics and the diagnosis of pulmonary aspergillosis based on the revised EORTC/MSG criteria are shown in Table 1.

In the proven and probable IPA category, 22.2% (4 of 18) of patients had malignant blood system diseases, 38.9% (7 of 18) of patients had chronic respiratory disease, and 33.3% (6 of 18) of patients received immunosuppressive drugs or corticosteroids therapy. Among the seven IPA patients with chronic respiratory diseases, 71.4% (5 of 7) of patients used immunosuppressive drugs or corticosteroids. The one patient in the proven category had no underlying disease. The mortality rate was 44% (8 of 18) for IPA patients. 72% (13 of 18) of IPA patients used the antifungal agent voriconazole. One IPA patient was coinfected with* Pneumocystis jirovecii* pneumonia (PCP), one patient was culture-positive for* Candida sp.* in the BALF, and one patient was culture-positive for* Candida sp.* in the sputum.

Three of the four CNA patients had underlying pulmonary tuberculosis, and one patient had diabetes mellitus. All five ABPA patients had no underlying disease. One patient with prior aspergilloma was admitted and diagnosed with aspergilloma coinfected with a bacterial infection.

### 3.2. Performance of BALF-GM and Serum-GM Assays

The median index of the BALF-GM assay in patients with IPA was 1.909 (interquartile range (IQR), 0.915–3.859), by contrast, the median index of the serum-GM assay was 1.072 (IQR, 0.634–4.194) (*P* > 0.05). In the patients of pulmonary aspergillosis excluded IPA, the median index of the BALF-GM assay was 1.958 (IQR, 0.469–3.579), and that of serum-GM assay was 0.348 (IQR, 0.152–1.172) (*P* < 0.05). Furthermore, the median index of the BALF-GM assay in patients with nonpulmonary aspergillosis was 0.421 (IQR, 0.246–0.756), whereas that of the serum-GM assay was 0.208 (IQR, 0.165–0.343) (*P* < 0.001). The sensitivity, specificity, PPV, and NPV of both assays are shown in Table 2. At a diagnostic cutoff index of 0.5, the sensitivity of the BALF-GM assay was higher than that of the serum-GM assay (85.7% versus 67.9%, *P* < 0.05), but the specificity of the BALF-GM assay is lower than that of the serum-GM assay (62.4% versus 93.5%, *P* < 0.05). As the cutoff index increased, the specificity of the BALF-GM assay increased, with a corresponding detriment of sensitivity. Additionally, the sensitivity of the BALF-GM assay was superior to that of fungal culture (85.7% versus 50%, *P* < 0.05).

**Figure 1:**
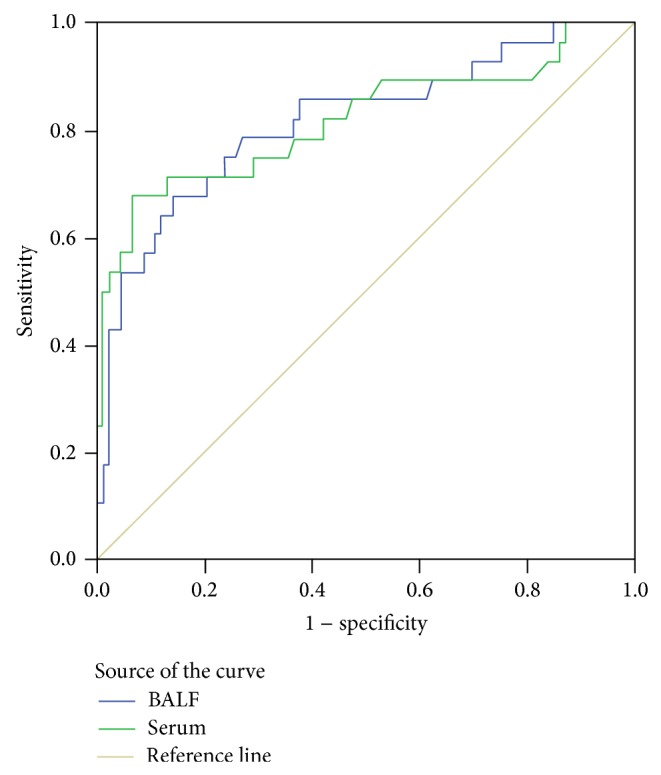
The ROC curves of the BALF-GM and serum-GM assays for the diagnosis of invasive aspergillosis and noninvasive aspergillosis.

The areas under the ROC curves were 0.817 (95% CI: 0.718–0.916) for BALF-GM and 0.819 (95% CI: 0.712–0.926) for serum-GM (Figure 1). The optimal cutoff index for the BALF-GM assay was 1.19. At this cutoff index (1.19), the sensitivity, specificity, PPV, and NPV were 67.9%, 89.2%, 65.5%, and 90.2%, respectively. The optimal cutoff index for the serum-GM assay was 0.55.

At the cutoff index of 1.19 (BALF-GM) and 0.55 (serum-GM), 72.2% (13 of 18) of IPA patients were BALF-GM positive and 83.3% (15 of 18) of IPA patients were serum-GM positive. Four of the CNA patients were BALF-GM positive, and two of the four patients were serum-GM positive. One of the five ABPA patients was BALF-GM positive, and two were serum-GM positive. The one patient with pulmonary aspergilloma was BALF-GM positive but serum-GM negative.

### 3.3. False Positivity of the BALF-GM Assay

A total of 35 patients with nonpulmonary aspergillosis had a BALF-GM index over 0.5 (Table 3). When the cutoff index of the BALF-GM assay was increased to 1.19, the false positive rate dropped from 37.6% to 14.0% compared with the cutoff index of 0.5 (*P* < 0.05).

## 4. Discussion

We identified 18 cases of proven and probable IPA in this retrospective study, of which 38.9% had underlying chronic respiratory disease and 71.4% had received immunosuppressive drugs or corticosteroids, which indicated the possibility of these patients being infected with IPA.

A meta-analysis conducted by Leeflang et al. [8] reported a sensitivity of 78% (95% CI: 61%–89%) and a specificity is 81% (95% CI: 72%–88%) for the serum-GM assay. Our study demonstrated a sensitivity of 67.9% and a specificity of 93.5% for the serum-GM assay used for the diagnosis of IPA. Additionally, our study showed that serum-GM assay has an optimal diagnostic value at the cutoff index of 0.55 in the population under our study, which is similar to the cutoff index of 0.50 in the clinic. The GM levels in the serum were relevant to both the fungal loads and the immune states of the patients. The GM levels released into the blood are quite low at the early stages of IPA, when the vessels are only mildly invaded; besides, the application of antifungal agents can decrease GM levels in the blood. In a host with normal immunity, the GM levels are rapidly eliminated [9]. All these factors collectively explain the inability of the serum-GM assay to detect IPA early with excellent sensitivity.

BALF can be directly lavaged from the lung, which is target organ for pulmonary aspergillosis. As a result, the BALF-GM assay offers an early and more sensitive detection of GM than the serum-GM assay for the diagnosis of IPA. Zou et al. [10] performed a meta-analysis on the diagnostic efficiency of the BALF-GM assay based on over 30 studies, which showed that the sensitivity of the BALF-GM assay was 87% (95% CI: 79%–92%), and the specificity was 89% (95% CI: 85%–92%). Similarly, Meersseman et al. [11] reported a sensitivity and specificity of 87% and 88%, respectively, for the diagnosis of IPA in patients admitted to the intensive care unit (ICU). Our findings are in agreement with that of previous studies, with the BALF-GM assay showing a higher sensitivity than the serum-GM assay (85.7% versus 67.9%, *P* < 0.05).

Nguyen et al. [12] compared the performance of the BALF-GM and serum-GM assay in four cases of noninvasive pulmonary aspergillosis and found that the BALF-GM assay has a higher positivity rate than the serum-GM assay. Kono et al. [13] showed that the sensitivities of the BALF-GM and serum-GM assays were 85.7% and 14.3% for diagnosing CNA and ABPA, respectively. Park et al. [14] found that the sensitivity was 92% for BALF-GM, but only 38% for serum-GM. In our present study, we report a BALF-GM assay sensitivity of 80% (8 of 10 cases) and a serum-GM assay sensitivity of 40% (4 of 10 cases) for the diagnosis of noninvasive pulmonary aspergillosis, suggesting that the BALF-GM assay is more efficient in diagnosing this infection than the serum-GM assay.

Various factors can lead to false positivity in the BALF-GM assay. One such factor is the administration of *β*-lactam antibiotics, especially piperacillin-tazobactam or amoxicillin and clavulanate, which was thought to be the reason causing false positives in the serum-GM assay [12, 14]. However, Penack et al. [15] did not consider piperacillin-tazobactam to be related with false positivity in the serum-GM assay. Furthermore, Nguyen et al. [12] found a high false positive rate with the BALF-GM assay in immunocompetent patients who did not receive piperacillin-tazobactam or amoxicillin and clavulanate potassium. In our study, we found that the BALF-GM false positive rates were not significantly different between patients who received *β*-lactam antibiotics and those who did not receive these drugs (Table 3).

Another important reason for the higher false positive rates with the BALF-GM assay is the contamination of the* Aspergillus* conidia in the respiratory tract or* Aspergillus* colonization. Garnacho-Montero et al. [16] reported that 50% of the isolates positive for* Aspergillus* from ICU patients were due to colonization. The patients in our study had underlying pulmonary disease, which may be another contributing factor for* Aspergillus* colonization, although the cultures from these patients were not positive.

We found that patients with* Pneumocystis jirovecii* pneumonia had a higher BALF-GM than patients with noninvasive pulmonary aspergillosis (*P* < 0.05). This finding is different from previous reports [17]. It is possible that these patients were more prone to* Aspergillus* colonization or were coinfected with* Aspergillus *spp.

The diagnostic cutoff index of the BALF-GM assay has been controversial due to the lack of proper standardization techniques. Hence, there is variation in the BALF volumes reported in different studies. The yield of BALF-GM is related to the lavage site; therefore, it is suggested to lavage the BALF on the lesion localized by computed tomography. Previous studies have considered a cutoff index higher than 0.5 for the BALF-GM assay. Our study demonstrated that the BALF-GM assay has better diagnostic efficiency at the cutoff index of 1.19, with a sensitivity of 67.9% and a specificity of 89.2%. We propose that the cutoff index for the BALF-GM assay should be defined comprehensively.

## 5. Conclusions 

BALF-GM assay was more sensitive in diagnosing invasive and noninvasive aspergillosis compared with the serum-GM assay and fungal cultures; however, the false positive rate was high. Additionally, the lavage technique for BALF and the diagnostic cutoff index for the BALF-GM assay need to be standardized, and factors inducing false positivity in this assay need to be determined.

## Figures and Tables

**Table 1:** Characteristics of patients enrolled in the study.

Total number	121 (100%)
Gender	
Male	62 (51.2%)
Female	59 (48.8%)
Age	
18–30	14 (11.6%)
31–40	7 (5.8%)
41–50	12 (9.9%)
51–60	28 (23.1%)
61–70	21 (17.4%)
71–80	24 (19.8%)
>80	15 (12.4%)
Average age	59.3
Basal conditions	
Possessing host factors	49 (40.5%)
COPD	11 (9.1%)
Others	61 (50.4%)
Diagnosis of aspergillosis	
Proven IPA	1 (0.8%)
Probable IPA	17 (14.0%)
CNA	4 (3.3%)
ABPA	5 (4.1%)
Possible IPA	9 (7.4%)
Aspergilloma	1 (0.8%)
Others	84 (69.4%)

**Table 2:** The diagnostic values of the BALF-GM and serum-GM assays at the different cutoff indices.

Cutoff	Sensitivity	Specificity	PPV	NPV
BALF-GM				
≥0.5	85.7%	62.4%	40.7%	93.5%
≥1	67.9%	79.6%	50.0%	89.2%
≥1.5	65.4%	92.5%	70.8%	90.5%
≥2	46.4%	96.8%	81.2%	85.7%

Serum-GM				
≥0.5	67.9%	93.5%	76.0%	90.6%
≥1	50.0%	98.9%	93.3%	86.8%
≥1.5	25.0%	98.9%	87.5%	81.4%
≥2	25.0%	100.0%	100.0%	81.6%

Culture				
	50.0%	98.9%	93.3%	86.8%

**Table 3:** Analysis of factors contributing to false-positives in the BALF-GM assay.

	False positive (*n* = 35)	Negative (*n* = 58)	*P*
Age	60 (26–86)	59 (20–92)	0.609
Gender (male/female)	18/17	29/29	0.894
Host factors	15 (42.8%)	29 (50.0%)	0.367
Cefepime	5 (14.3%)	4 (6.9%)	0.289
Cefoperazone-sulbactam	7 (20.0%)	13 (22.4%)	0.784
Piperacillin-sulbactam	2 (5.7%)	2 (3.4%)	0.630
Piperacillin-tazobactam	2 (5.7%)	4 (6.9%)	1
Meropenem	4 (11.4%)	6 (10.3%)	1
Fluoroquinolones	10 (28.6%)	8 (13.8%)	0.081
*Candida sp.* colonization	8 (22.9%)	9 (15.5%)	0.375
Coinfected with *Pneumocystis jirovecii *	7 (20%)	2 (3.4%)	0.024
